# Vivid biofluorescence discovered in the nocturnal Springhare (Pedetidae)

**DOI:** 10.1038/s41598-021-83588-0

**Published:** 2021-02-18

**Authors:** Erik R. Olson, Michaela R. Carlson, V. M. Sadagopa Ramanujam, Lindsay Sears, Sharon E. Anthony, Paula Spaeth Anich, Leigh Ramon, Alissa Hulstrand, Michaela Jurewicz, Adam S. Gunnelson, Allison M. Kohler, Jonathan G. Martin

**Affiliations:** 1 Departments of Environmental Sciences and Natural Resources, Northland College, 1411 Ellis Avenue, Ashland, WI 54806 USA; 2 Department of Preventive Medicine and Population Health, The University of Texas Medical Branch, Galveston, TX 77555 USA; 3 Omaha’s Henry Doorly Zoo and Aquarium, 3701 South 10th Street, Omaha, NE 68107 USA; 4 Mesker Park Zoo and Botanic Garden, 1545 Mesker Park Drive, Evansville, IN 47720 USA; 5 Natural Resources Ecology Laboratory, Colorado State University, Fort Collins, CO 80523 USA

**Keywords:** Evolutionary ecology, Biochemistry, Evolution

## Abstract

Biofluorescence has been detected in several nocturnal-crepuscular organisms from invertebrates to birds and mammals. Biofluorescence in mammals has been detected across the phylogeny, including the monotreme duck-billed platypus (*Ornithorhyncus anatinus*), marsupial opossums (Didelphidae), and New World placental flying squirrels (*Gluacomys* spp.). Here, we document vivid biofluorescence of springhare (Pedetidae) in both museum specimens and captive individuals—the first documented biofluorescence of an Old World placental mammal. We explore the variation in biofluorescence across our sample and characterize its physical and chemical properties. The striking visual patterning and intensity of color shift was unique relative to biofluorescence found in other mammals. We establish that biofluorescence in springhare likely originates within the cuticle of the hair fiber and emanates, at least partially, from several fluorescent porphyrins and potentially one unassigned molecule absent from our standard porphyrin mixture. This discovery further supports the hypothesis that biofluorescence may be ecologically important for nocturnal-crepuscular mammals and suggests that it may be more broadly distributed throughout Mammalia than previously thought.

## Introduction

As our understanding of the visual capacities of various species develops^1,2^, we are realizing that many species are capable of seeing the world through a different lens, and that, in addition to or as an alternative to visible light (approximately, 400–700 nm), other wavelengths of light may be ecologically important. For example, some species use cues from ultraviolet (UV) light for sexual selection^3^, predator avoidance^4^, and foraging^5^. Many mammals are sensitive to UV wavelengths of light^1,2,6^, especially marsupials^7^ (New World opossums), a monotreme^8^ (platypus, *Ornithorhynchus anatinus*), and some rodents^7^ (Sciurognathi).

Biofluorescence, the absorption of short wavelengths of light and re-emission of longer wavelengths of light, has been increasingly observed in a wide range of invertebrates^9^, fishes^10,11^, reptiles and amphibians^12–16^ and birds ^3,17,18^. Within mammals, biofluorescence has been observed in New World placental flying squirrels^19^ (*Glaucomys* spp.), New World marsupial opossums^20,21^ (Didelphidae), and the monotreme duck-billed platypus (*O. anatinus*) of Australia and Tasmania^22^. These groups, inhabit three continents and a diverse array of ecosystems and are widely distributed across the mammalian family tree. All of these biofluorescent mammalian species are nocturnal-crepuscular^19–22^. Thus, biofluorescence in mammals may be of significance to nocturnal-crepuscular mammals that are active in low light environments^9,22^. Here we document biofluorescence in another nocturnal mammal, springhare (Pedetidae)—the first well-documented biofluorescence of an Old World eutherian mammal.

Like other biofluorescent mammals, springhares (Pedetidae) are nocturnal. Apart from their nocturnal lifestyle, springhares are not ecologically similar to other known fluorescent species and are rather distant relatives of the biofluorescent rodents within Sciuridae^19,23^. Both species of springhares are endemic to Africa: the springhare (*Pedetes capensis*) lives in southern Africa; and the East African springhare (*P. surdaster*) inhabits portions of Kenya and Tanzania^23^. Both species are nocturnal, fossorial grazers that inhabit semi-arid areas^24,25^. Springhares are mainly solitary, sheltering in their own burrows during the day and preferentially foraging individually in short-grass environments at night^24^. Although short-grass environments provide springhares with minimal cover from predators, like mongooses (Herpestidae) and jackals (Canidae), these open areas provide springhares with clear lines of sight for the detection of predators and few obstacles to escape via explosive, bipedal leaps^25^. Here we document vivid biofluorescence in Pedetidae, its variation, its underlying chemical nature, and its potential ecological implications.

## Results

While studying biofluorescence in New World flying squirrels (*Glaucomys* spp.) and members of Anomaluromorpha at the Field Museum of Natural History (FMNH) in Chicago, Illinois, USA, in April 2018^19^ and November 2019^22^, we discovered springhare biofluorescence and subsequently documented the trait in multiple specimens and captive individuals.

**Figure 1:**
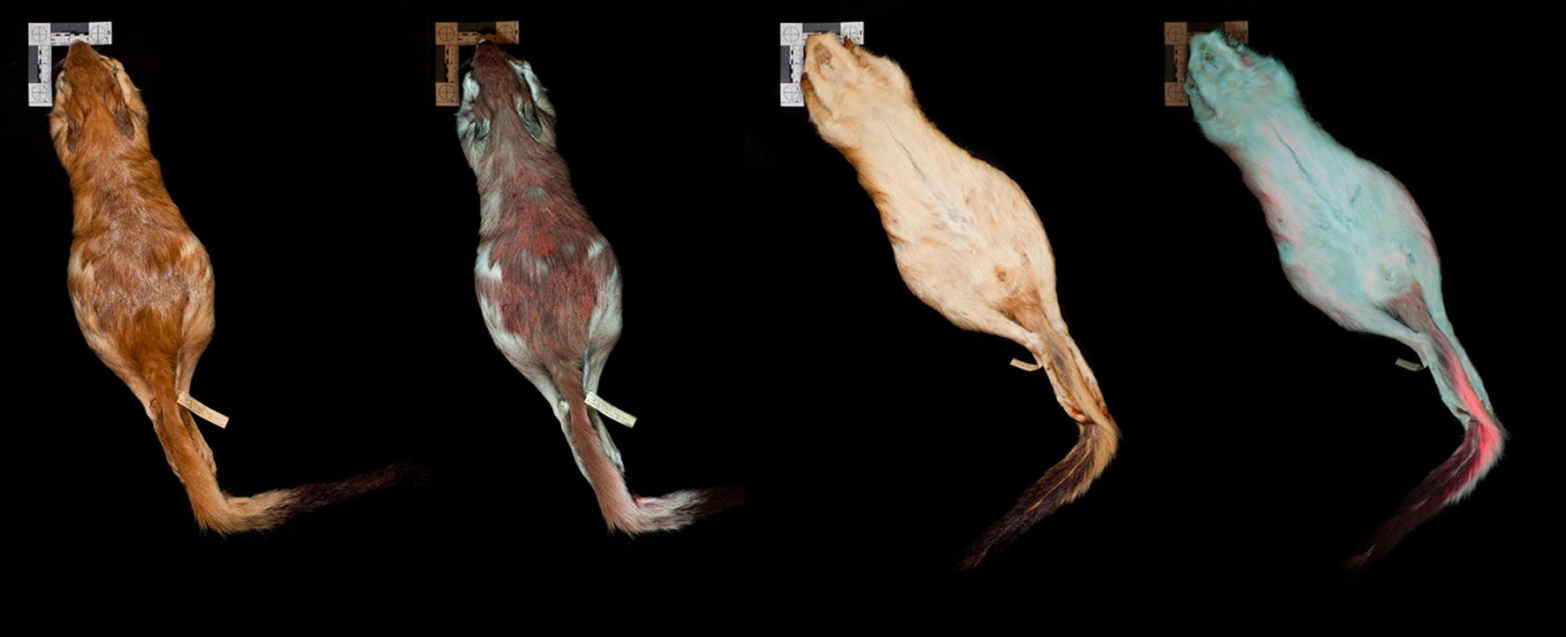
Biofluorescence in East African springhare museum specimens. A male *Pedetes surdaster* museum specimen (FMNH 73067) collected from the Central Province of Kenya in 1952, photographed under visible light (first and third image from left) and 395 nm ultraviolet light using a 470 nm longpass filter. Orange to red biofluorescence (~ 650 nm) is observed on the dorsal (leftmost images) and ventral surfaces (rightmost images) of this museum specimen from the Field Museum of Natural History (FMNH) in Chicago, Illinois, USA.

**Figure 2:**
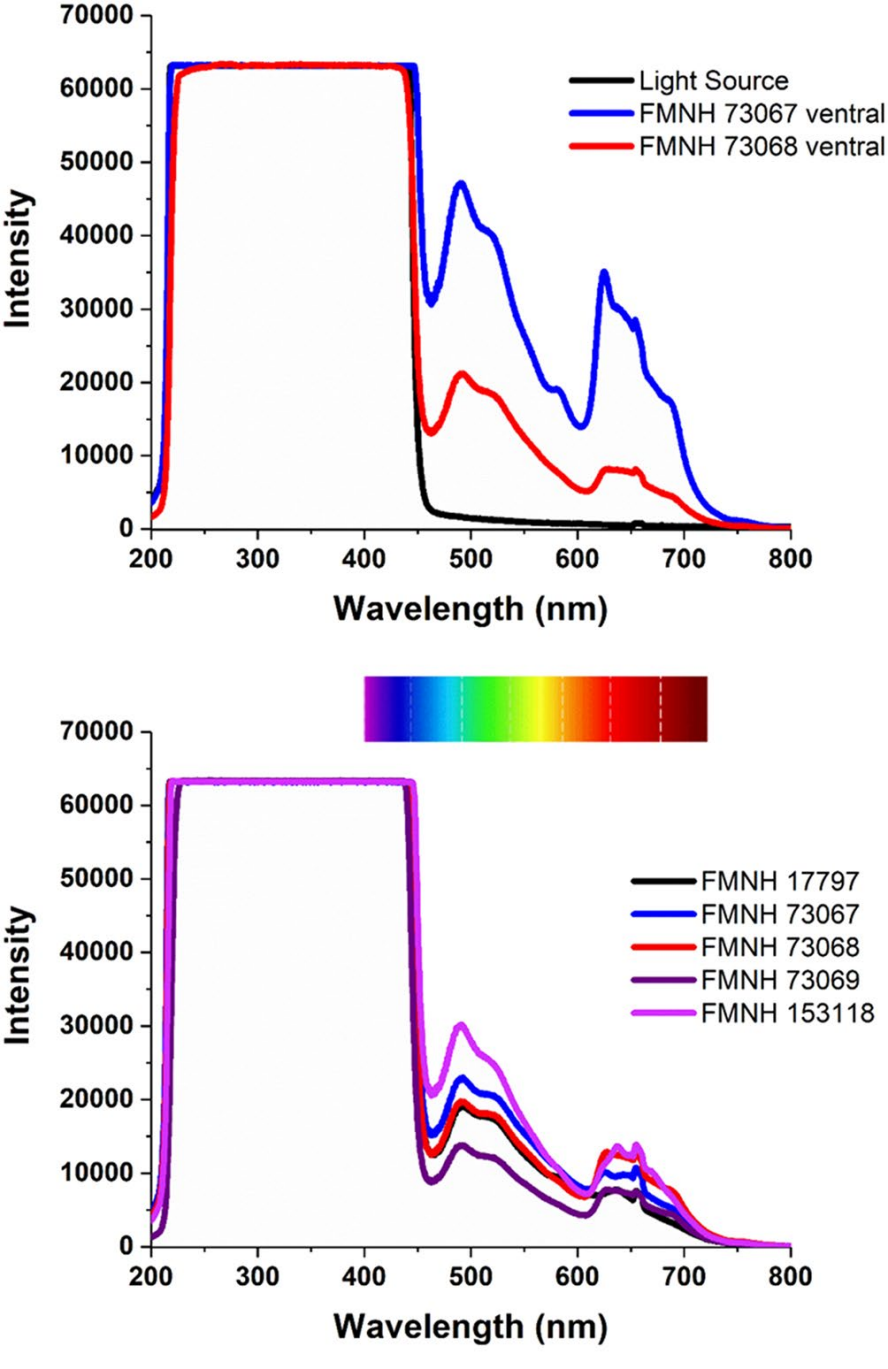
Spectra of biofluorescence observed in East African springhare. (Top panel) Spectra of biofluorescence on the ventral side of two different *P. surdaster* specimens (red and blue lines) in comparison to the spectrum of the light source taken against a diffuse reflectance standard (black line). (Bottom panel) Spectra of biofluorescence from the dorsal side of five different *P. surdaster* specimens. Peaks in the visible range (400–750 nm) represent biofluorescence.

We examined a total of 14 museum specimens, including eight museum specimens of *P. capensis* collected from Angola (*n* = 2) and Botswana (*n* = 6), and six specimens of *P. surdaster* collected from Kenya (*n* = 3) and Tanzania (*n* = 3) (Fig. 1, Supplementary Table S1). We examined individuals of both sexes, including four males (*P. capensis, n* = 2; *P. surdaster, n* = 2) and ten females (*P. capensis, n* = 6; *P. surdaster, n* = 4), and specimen collection dates ranged from 1905 to 1963. For museum specimens, we observed and photographed biofluorescence on both the dorsal and ventral side of each specimen following the methods of Anich et al.^22^ (Canon EOS 50D, Canon USA Inc., Melville, NY, USA; Sigma 17–70 mm f 2.8–4 DC Macro) under visible light (Canon Speedlite 430EX) and then separately under 395 nm UV light (LED UV flashlight, iLumen8 100 LED). We photographed a subset of specimens using a 470 nm longpass filter (K&F Concept, Guangdong Sheng, China; Tiffen Yellow 2 #8, Hauppauge, New York, USA) under UV illumination to absorb any residual light in the blue wavelengths. We also captured UV reflectance using a Nurugo SmartUV camera (Union community Co., Ltd., Seoul, Republic of Korea), which suggested that very little UV light was being absorbed on either dorsal or ventral surfaces of a subset of springhare specimens. Following the methods of Anich et al.^22^ for fluorescence spectroscopy (Ocean Optics USB2000^+^, Largo, Florida, USA), we identified two peaks of fluorescence at 500 and 650 nm for a section of highly fluorescent fur on the ventral surface (Figs. 1 and 2).

**Figure 3:**
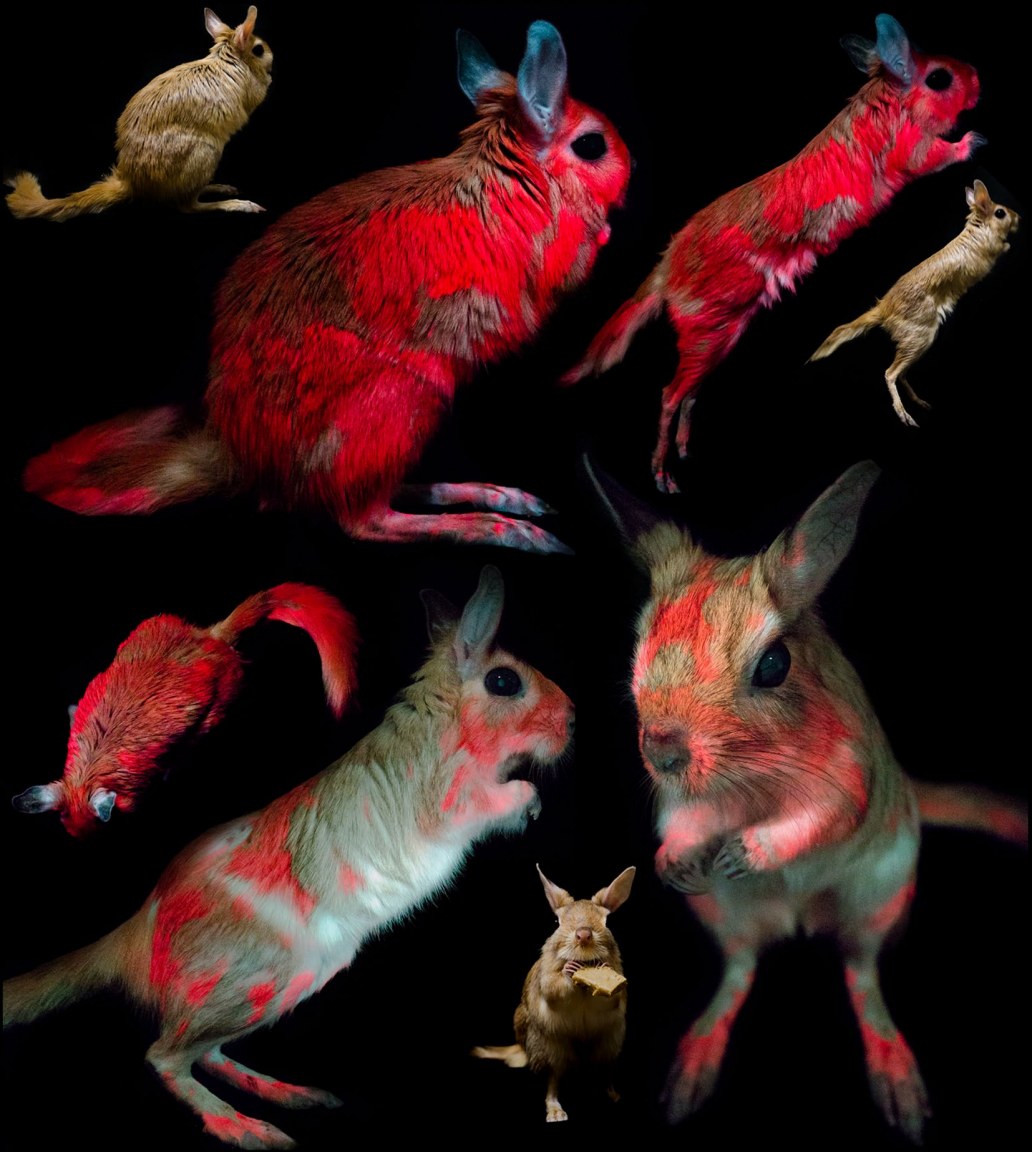
Biofluorescence in captive springhare. *Pedetes capensis* photographed under visible light (three insets) and under 395 nm ultraviolet light with a 470 nm longpass filter to documenting the orange to red biofluorescence (~ 650 nm) of springhare. Images contain two different captive-bred, captive individuals (Individual #4, female, bottom two larger images under ultraviolet light; Individual #2, male, all remaining images) from the Omaha’s Henry Doorly Zoo & Aquarium in Omaha, Nebraska, USA.

**Figure 4:**
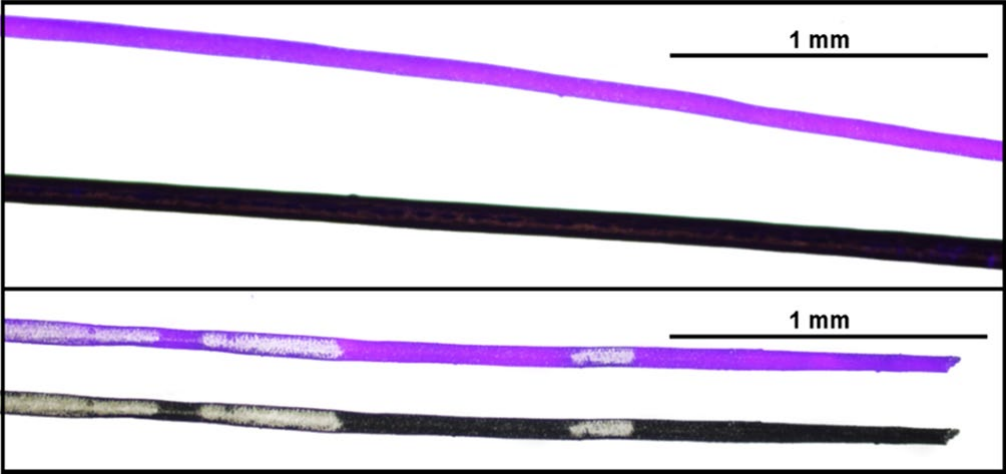
Biofluorescent hair of springhare. (Top panel) Biofluorescent hair of *Pedetes capensis* compared to the non-biofluorescent hair of a human photographed under 395 nm ultraviolet light at 4× magnification with a 100 ms exposure, and (bottom panel) a single strand of *P. capensis* hair photographed under 395 nm ultraviolet light and visible light at 4× magnification with a 100 ms exposure; highlighting variation observed in hair pigmentation and fluorescence.

We also observed and photographed biofluorescence on five living captive-bred *P. capensis* individuals (three males and two females) at Omaha’s Henry Doorly Zoo and Aquarium in Omaha, Nebraska and one deceased (due to natural causes) individual (female) at the Mesker Park Zoo & Botanic Garden in Evansville, Indiana (Fig. 3; Supplementary Table S2, Canon EOS 6D, Canon USA Inc., Melvill, New York, USA; Canon 17–40 mm f 2.8–4; LED 395 UV flood light; Tiffen Yellow 2 #8, Hauppauge, New York, USA; white balance corrected from DGK Color Tools WB card, DGK Color Tools LLC, Waban, Massachusetts, USA). Using compound light microscopy (Eclipse E2300, with DSFI2 camera, Nikon Corporation, Tokyo, Japan), we examined hair samples from the recently deceased captive individual springhare under visible and UV light with 4× magnification. We photographed a human hair under the same conditions for comparison. We observed fluorescence of individual springhare hair fibers and variation in the presence of fluorescence within individual hair fibers, suggesting that the fluorescence is distributed through the thickness of the cuticle and absent from the core and tips of hair fibers (Fig. 4). Washing the hair or fur with Dawn dish soap (Cincinnati, Ohio, USA) did not remove or diminish fluorescence or result in a transfer of the fluorescence.

**Figure 5:**
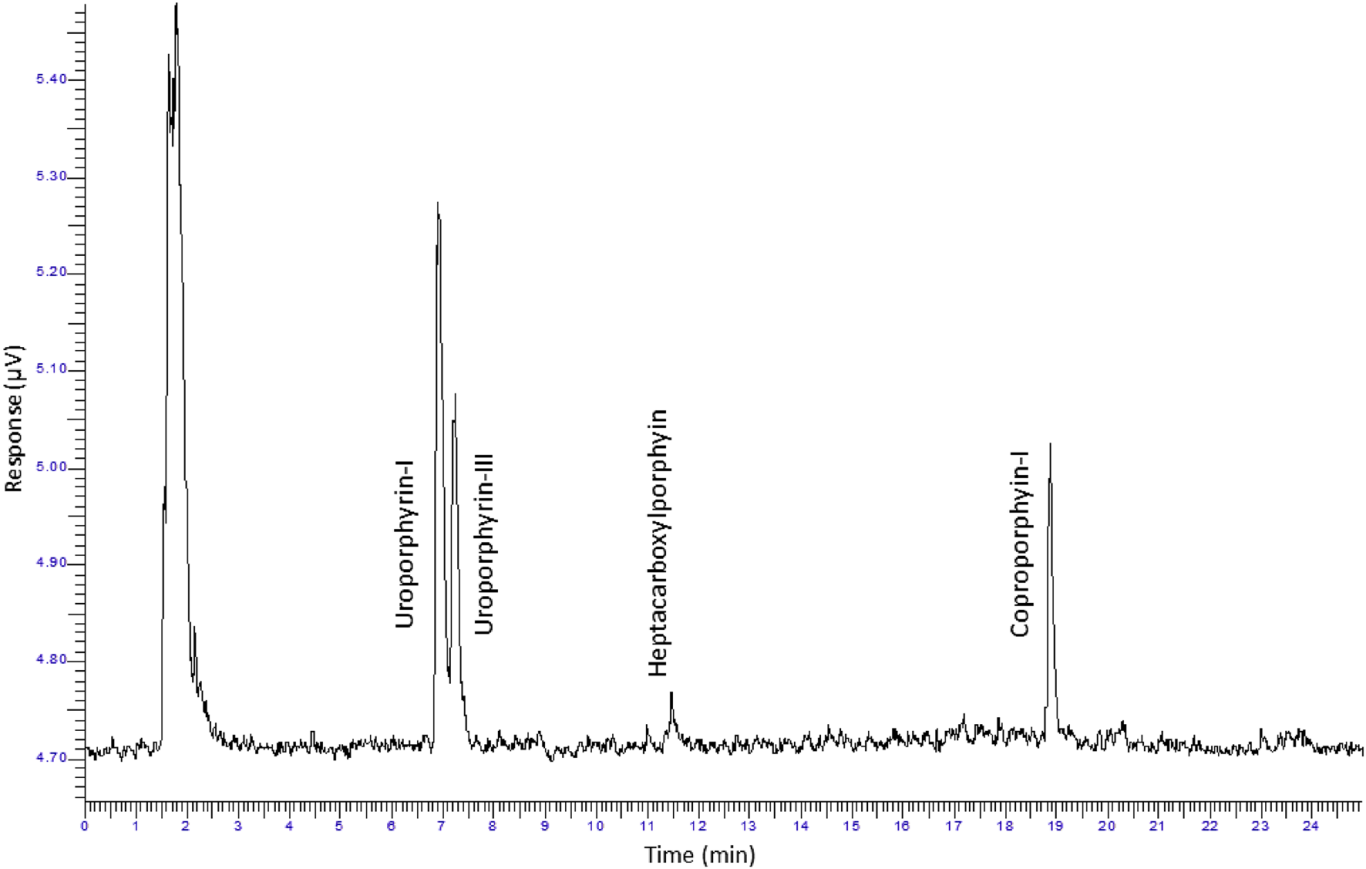
Identification of the porphyrins responsible for biofluorescence in Pedetidae. High performance liquid chromatograph of extracted pigment from captive *Pedetes capensis* fur. The response times in this sample were compared to that of a standard mixture of porphyrins (Supplementary Fig S1) which allowed for identification of uroporphyrin-I, uroporphyrin-III, heptacarboxylporphyrin, and coproporphyrin-I in the sample. The peak centered around two minutes has not been assigned and does not correspond to any of the porphyrins in the standard mix (uroporphyrin, heptacarboxylporphyrin, hexacarboxylporphyin, pentacarboxylporphyrin, coproporphyrin, and mesoporphyrin).

We used thin layer chromatography to separate fluorescent extracts from hair samples. Using high performance liquid chromatography (HPLC), we revealed the presence of fluorescent porphyrin species including uroporphyrin-I, uroporphyrin-III, heptacarboxylporphyrin, hexacarboxylporphyrin, and coproporphyrin-I (Fig. 5; Supplementary Figs S1 and S2). We also detected an unassigned molecule absent from our standard mixture of porphyrins that peaked at approximately two minutes, which may also play a role in the biofluorescence observed (Fig. 5; Supplementary Figs S1 and S2).

For *P. capensis* and *P. surdaster*, all individuals exhibited orange to red biofluorescence, although we did observe variation in the intensity of biofluorescence across individuals. Biofluorescence was pronounced on both the dorsal and ventral surfaces (Fig. 1), and fluorescence intensity for the dorsal surfaces was often more intense in the head and posterior region (Figs. 1 and 3). Ventrally, biofluorescence occurred predominantly along the inner thigh and tail (Fig. 1). We observed notable patchiness in the biofluorescence in both museum and captive specimens (Figs. 1 and 3).

## Discussion

We have discovered a funky and vivid porphyrin-based biofluorescence in Pedetidate, representing the first well-documented biofluorescence of an Old World eutherian mammal. While we do not have a large enough sample size to draw conclusions about the frequency of this trait in wild populations, we did consistently observe the trait in six captive individuals, as well as, 14 museum specimens collected at different times over a 58-yr. period and across seven separate locations in four countries (Fig. 3). Due to the spatial, temporal, and contextual (i.e., captive or wild) diversity of our specimens, we suspect this trait is not environmental. Both male and female specimens fluoresced in the same regions and with the same intensity, generally; therefore, we suspect that the trait is not sexually dimorphic. The fact that biofluorescence was not easily removed via washing and was present on museum specimens from 1905 suggests that the biofluorescence is a part of the physical anatomy of the hair fibers for Pedetidae. Biofluorescence appeared more vivid in living individuals than in museum specimens, potentially indicating some degradation over time (Figs. 1 and 3).

We detected multiple species of porphyrin in extracts from springhare hair samples. Porphyrin-based biofluorescence has been suspected or confirmed in many marine invertebrates^26,27^, the plumage of many bird species^18,28–30^, the bones of at least one species of rodent^31^, and at least one species of Platyhelminthes^9^ (*Platydemus manokwari*). Here we detected uroporphyin-I, -III, heptacarboxylporphyrin, hexacarboxylporphyrin, coproporphyrin-I, and one unassigned molecule absent from our standard porphyrin mixture. The isolated porphyrins are formed by oxidation of porphyrinogens, which are intermediates in the biosynthetic pathway of heme^32^. Uroporphyrin and coproporphyrin fluoresce between 570 and 720 nm in various conditions^33,34^, suggesting that at least both uroporphyrin and coproporphyrin play a role in causing biofluorescence in springhare. We recommend future studies be done to determine whether this biofluorescence is an advantageous evolutionary trait or a disease, such as porphyrias as seen in fox squirrels (*Sciurus niger*)^31,35,36^, canefield rats (*Rattus sordidus*)^37^, and humans (*Homo sapiens*)^32,38^.

Biofluorescence in both species of *Pedetes* was notably patchy (Figs. 1 and 3), and observations of captive individuals indicated that the areas most impacted by grooming and intra-specific interactions, i.e., reproduction, appear to overlap relatively strongly with areas most consistently exhibiting biofluorescence; this would suggest that the fluorescence might be externally applied to the fur during certain behaviors. However, thorough washing of the fur did not result in the removal or transfer of the fluorescence, and observations of enclosures of captive individuals did not reveal any transferred fluorescence. Additionally, UV images of a single live individual taken 14 months apart indicate that the individual patterns are relatively consistent, further suggesting that an alternative explanation for the patchiness of biofluorescence in springhare (Fig. 3) may exist. Springhares are predominantly solitary and tend to forage in more open areas with sparse vegetation and, therefore, have greater exposure to predators due to the lack of cover or group vigilance. Thus, we hypothesize that the patchiness of biofluorescence in springhares could function as a camouflage of sorts, but this would depend on the UV sensitivity of their predators. We recommend additional behavioral and biological research to further understand the potential ecological relevance of springhare biofluorescence and patchiness thereof.

The ecological implications of biofluorescence in springhare remain unknown. However, like other biofluorescent animals, springhares are nocturnal. Biofluorescence in mammals has been detected mainly in nocturnal-crepuscular^19–22^ and UV-sensitive^7,39^ species, and UV-color vision appears to be ecologically important to many nocturnal-crepuscular mammals^1^. While we cannot determine why Pedetidae exhibits biofluorescence, our observations add further support for the hypothesis that biofluorescence and UV wavelengths of light may be ecologically important for nocturnal-crepuscular mammals^1,9,19,22^. Our observations also suggest that biofluorescence may be more broadly distributed throughout Mammalia than previously thought^22^.

## Methods

All methods were carried out in accordance with the relevant guidelines and regulations. Protocol for captive animal observation and photography was evaluated and considered exempt from the Omaha’s Henry Doorly Zoo & Aquarium’s ACUC. Protocol for hair collection from the deceased (due to natural causes) individual was evaluated by Mesker Park Zoo & Botanic Garden Research Committee and considered exempt from the Mesker Park Zoo & Botanic Garden ACUC. Protocol for the microscopy and photography of a human hair was evaluated and considered exempt from the Northland College IRB. Informed consent was obtained for the microscopy and photography of the strand of human hair.

### Fluorescence spectroscopy

We used an Ocean Optics Flame-S-UV-VIS-ES (Largo, Florida, USA) in fluorescence mode (integration time = 1 s, and 5 scans per spectrum) and an Ocean Optics DH-2000-BAL deuterium light source (Largo, Florida, USA) to take fluorescence spectra. We selected an intense fluorescent spot on a subset of the Pedetidae specimens and placed a probe holder directly on that spot with the probe at 45° relative to the sample. Fluorescence spectra were taken at five different places within that spot, and these five spectra were averaged. We recorded the light source spectrum against a polytetrafluoroethylene diffuse reflectance standard.

### Extraction of biofluorescent compounds

We collected hair samples from the recently deceased captive individual of *P. capensis* at the Mesker Park Zoo & Botanic Garden. We attempted to determine if the fluorescence could be removed via washing with Dawn dish soap (Cincinnati, Ohio, USA). We combined samples of fluorescent fur (washed and unwashed) into two samples (68.8 mg and 95.1 mg for sample 1 and sample 2, respectively), and each sample was refluxed in a 25 mL round bottom flask, with 1 mL of 0.5 M NaOH until all hair was dissolved and the solvent appeared pink under UV light (approx. 16 min). We separated the solution using thin layer chromatography and a mixture of 4 mL DMF, 35 mL MeOH, 6 mL ethylene glycol, 0.4 mL glacial acetic acid, 18 mL 1-chlorobutane, and 20 mL CHCl_3_ as the solvent^40^. The thin layer chromatography plates had aluminum backs and were coated with silica gel 60 F_254_. After separation, three distinct pink bands were visible under a handheld UV light. We collected and washed the pink silica with acetone and deionized water until the silica was no longer pink. Then, we filtered and reduced the extracts under pressure until a yellow oil remained which was sent for HPLC analysis.

### Characterization of fluorescent compounds

We used a Perkin Elmer Series 200 HPLC instrument (Waltham, MA, USA) connected in series with a C18 reversed phase Onyx Monolithic HD-C18 liquid chromatography column (100 mm length × 4.6 mm internal diameter, Phenomenex Inc., Torrance, CA, USA), and a Perkin Elmer Series 200 fluorescence detector to characterize the extracts. All solvents were of HPLC grade and purchased from Fisher Scientific, Inc., USA. The components of mobile phase A (pH = 5.16) contained 800 mL of Milli-Q water mixed with 100 mL acetonitrile, 55 ml glacial acetic acid, and 45 ml concentrated ammonium hydroxide. The components of mobile Phase B contained 900 ml methanol mixed with 100 ml acetonitrile.

We dissolved extracted materials in 1 mL of 1 M hydrochloric acid, centrifuged and transferred the supernatant to amber colored auto-sampler vials. For the identification of porphyrin carboxylic acids, we used a chromatographic marker kit containing 5 micromole mixture of each octa-, hepta-, hexa-, penta-, tretra-, and di-carboxyl porphyrin acids (Frontier Scientific, Inc., Salt Lake City, UT, USA). Octa- and tetra-carboxyl porphyrin acids are conventionally called as Uropophyrin and Coproporphyrin, respectively. We dissolved the standards mixture of porphyrins in the tube in 10 mL of 1 M hydrochloric acid (High Purity grade from Fisher Scientific, Inc., Salt Lake City, UT, USA), and considered this mix as the HIGH standards mix. We created a ten times dilution of the HIGH standards mix and considered this the LOW standards mix.

We used a gradient elution program with an injection volume set to 100 µl and total run time 36 min^41,42^ (Table 1). Two vials of porphyrin carboxylic acid of the (HIGH and LOW concentrations) standards were also analyzed with each batch of samples. We identified the HPLC peaks in samples by matching retention times (min) of sample peaks with carboxyl porphyrin acid peaks (Supplementary Fig S1).

**Table 1:** Gradient elution program for high-performance liquid chromatography analysis of extracts from hair samples to determine the basis of biofluorescence in springhare (*Pedetes capensis*).

Step	Time (min)	Flow (ml/min)	Mobile phase A	Mobile phase B	Curve
0	2.0	1.20	100	0	0.0
1	2.0	1.20	85	15	6.0
2	18.0	1.20	0	100	2.0
3	5.0	1.20	100	0	2.0

## Supplementary Information

Supplementary Information

## Data Availability

All data produced in this study are included in the text and supplementary information documents.
