# Supplementary material for: Vivid biofluorescence discovered in the nocturnal Springhare (Pedetidae)
Source: Sci Rep. 2021 Feb 18;11:4125. doi: 10.1038/s41598-021-83588-0 (PMC7892538; doi:10.1038/s41598-021-83588-0)
Supplement: Supplementary file 1 — Supplementary Information [file 41598_2021_83588_MOESM1_ESM.docx]

**SUPPLEMENTARY INFORMATION**

**Vivid biofluorescence discovered in the nocturnal Springhare (Pedetidae)**

Erik R. Olson^1^*, Michaela R. Carlson^1^, V.M. Sadagopa Ramanujam^2^, Lindsay Sears^3^, Sharon E. Anthony^1^, Paula Spaeth Anich^1^, Leigh Ramon^4^, Alissa Hulstrand^1^, Michaela Jurewicz^1^, Adam S. Gunnelson^1^, Allison M. Kohler^1,5^, and Jonathan G. Martin^1^

^1^ Departments of Environmental Sciences and Natural Resources, Northland College, 1411 Ellis Avenue, Ashland, WI 54806, USA

^2^ Department of Preventive Medicine & Population Health, The University of Texas Medical Branch, Galveston, TX 77555, USA

^3^ Omaha’s Henry Doorly Zoo & Aquarium, 3701 South 10^th^ Street, Omaha, NE 68107, USA

^3^ Mesker Park Zoo & Botanic Garden, 1545 Mesker Park Drive, Evansville, IN 47720, USA

^5^ Natural Resources Ecology Laboratory, Colorado State University, Fort Collins, CO 80523, USA

*corresponding author; eolson@northland.edu

**Supplementary Table S1:** Museum specimens (including species, date of collection, locality, and sex) from the Field Museum of Natural History, Chicago IL, USA (FMNH) photographed and analyzed for biofluorescence

| Specimen | Species | Date collected | Country | State/Province | Sex |
| --- | --- | --- | --- | --- | --- |
| 38234 | Pedetes capensis | 4/17/1930 | Botswana | Ghanzi | Female |
| 38235 | Pedetes capensis | 4/20/1930 | Botswana | Ghanzi | Male |
| 38238 | Pedetes capensis | 4/20/1930 | Botswana | Ghanzi | Female |
| 38251 | Pedetes capensis | 6/2/1930 | Botswana | Ngamiland | Male |
| 38256 | Pedetes capensis | 7/16/1930 | Botswana | Kabulabula | Female |
| 38258 | Pedetes capensis | 7/15/1930 | Botswana | Kabulabula | Female |
| 84009 | Pedetes capensis | 12/13/1954 | Angola | Huila | Female |
| 84010 | Pedetes capensis | 12/13/1954 | Angola | Huila | Female |
| 73067 | Pedetes surdaster | 5/20/1952 | Kenya | Central Prov | Male |
| 153118 | Pedetes surdaster | 8/4/1963 | Kenya | Rift Valley Prov | Female |
| 73069 | Pedetes surdaster | 7/16/1952 | Tanzania | Mara Region | Male |
| 147621 | Pedetes surdaster | 8/17/1928 | Tanzania | Mara Region | Female |
| 17797 | Pedetes surdaster | 12/27/1905 | Kenya | Eastern Prov | Female |
| 73068 | Pedetes surdaster | 7/16/1952 | Tanzania | Mara Region | Female |

**Supplementary Table S2:** Captive individual *Pedetes capensis* (including house name, birthdate, status, locality, and sex) from the Omaha Henry Doorly Zoo & Aquarium in Omaha, Nebraska (OHDZA) and the Mesker Park Zoo & Botanic Garden in Evansville, Indiana (MPZ) photographed and observed for biofluorescence

| Individual | House name | Birthdate | Status | Locality | Sex |
| --- | --- | --- | --- | --- | --- |
| 1 | Snap | ~01 May-01 Dec 2011 | alive | OHDZA | male |
| 2 | Tumbleweed | 01 November 2014 | alive | OHDZA | male |
| 3 | Bluebell | 08 April 2016 | alive | OHDZA | male |
| 4 | Nettles | 07 May 2014 | alive | OHDZA | female |
| 5 | Poppy | 14 August 2009 | alive | OHDZA; MPZ | female |
| 6 | Daisy | 16 October 2002 | deceased* | OHDZA; MPZ | female |

*examined post-mortem


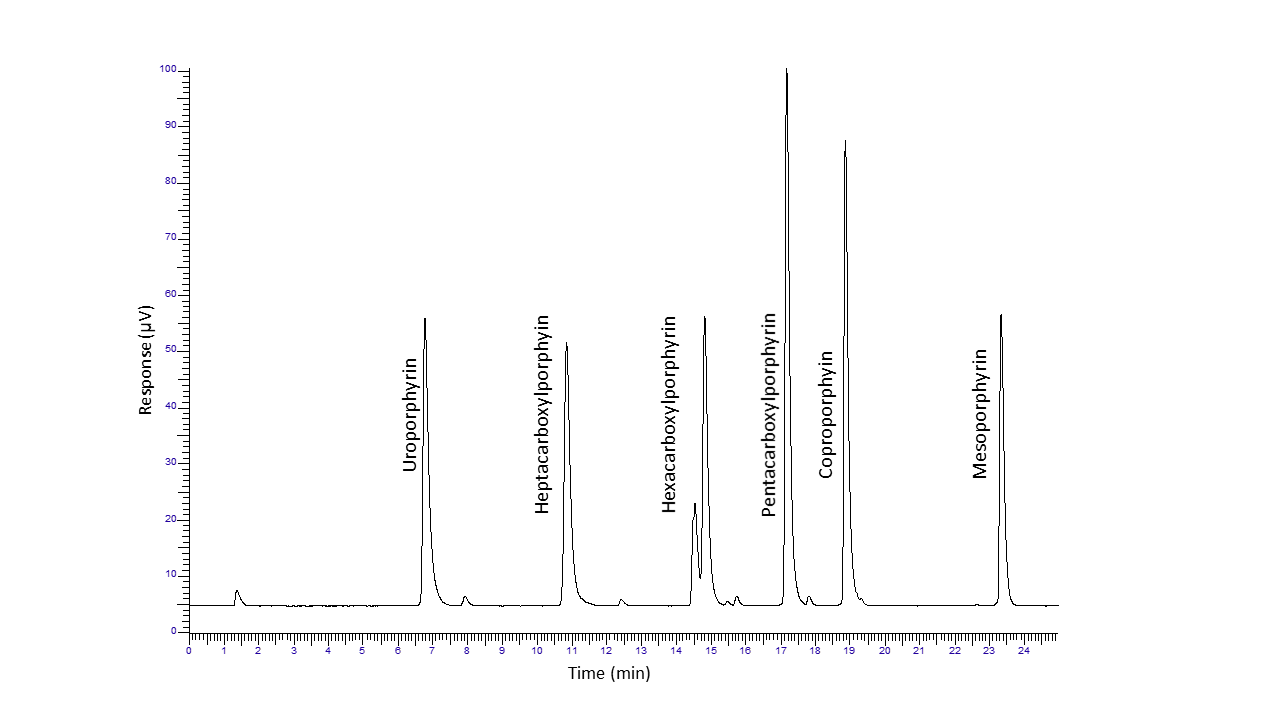


**Supplementary Figure S1:** Response times of porphyrins in standard mixture. High performance liquid chromatograph of a standard mixture of porphyrins (uroporphyrin, heptacarboxylporphyrin, hexacarboxylporphyin, pentacarboxylporphyrin, coproporphyrin, and mesoporphyrin). The response times for each of these porphyrins allowed for identification of peaks in the pigment extracts.


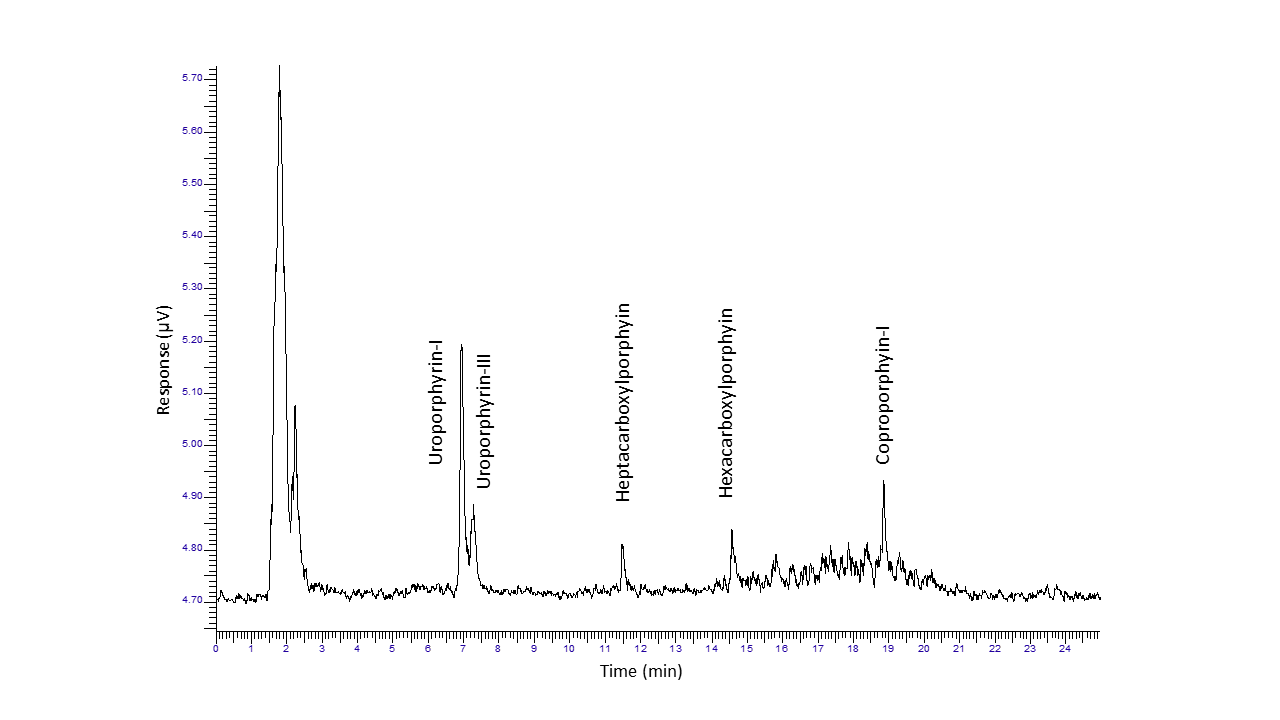


**Supplementary Figure S2:** Identification of the porphyrins responsible for biofluorescence in Pedetidae. High performance liquid chromatograph of a second batch of pigment extracted from *P.* *capensis* fur. Peaks were identified by comparison to the peaks from the standard mixture (Supplemental Fig S1). The chromatogram indicates that uroporphyrin-I, uroporphyrin-III, heptacarboxylporphyrin, hexacarboxylporphyrin, and coproporphyrin-I are present. The peak centered around 2 minutes has not been assigned but it does not correspond to any of the porphyrins in the standard mix (uroporphyrin, heptacarboxylporphyrin, hexacarboxylporphyin, pentacarboxylporphyrin, coproporphyrin, and mesoporphyrin).
